# 基于CSF3R突变与治疗后微小残留病对CEBPA双突变急性髓系白血病预后再分层研究

**DOI:** 10.3760/cma.j.issn.0253-2727.2022.12.008

**Published:** 2022-12

**Authors:** 龙 苏, 业辉 谭, 海 林, 薇 韩, 艳萍 杨, 晓亮 刘, 京男 孙, 秋菊 刘, 素君 高

**Affiliations:** 吉林大学第一医院血液科，长春 130021 Department of Hematology, the First Hospital of Jilin University, Changchun 130021, China

**Keywords:** 白血病，髓系，急性, CEBPA双突变, CSF3R突变, 微小残留病, 预后, Leukemia, myeloid, acute, CEBPA double mutations, CSF3R mutations, Measurable residual disease, Prognosis

## Abstract

**目的:**

研究CSF3R突变与治疗后微小残留病（MRD）在CEBPA双突变急性髓系白血病（AML）患者中的预后意义。

**方法:**

回顾性分析2012年1月至2018年9月就诊于吉林大学第一医院血液科的66例具有完整二代基因测序结果且进行了系列MRD监测的AML患者，研究治疗前CSF3R突变和治疗后MRD水平与患者治疗疗效及长期预后的相关性。

**结果:**

CSF3R突变患者的5年无复发生存（RFS）率和总生存（OS）率分别为15.2％和18.2％，明显低于无CSF3R突变患者的38.7％和60.6％（*P*值分别为0.006和0.038）。2个疗程化疗后MRD转阴患者的中位RFS与OS时间分别为64个月与未达到，明显长于MRD阳性患者的15个月和 48个月（*P*值分别为0.004和0.050）。Cox风险比例模型分析显示，CSF3R突变（*HR*＝0.317，95％*CI* 0.129～0.779，*P*＝0.012）、WT1突变（*HR*＝0.304，95％*CI* 0.115～0.804，*P*＝0.016）、NRAS突变（*HR*＝0.153，95％*CI* 0.061～0.385，*P*<0.001）为影响患者RFS的独立不良预后因素，CSF3R突变与MRD阳性趋向为OS的独立预后因素（*P*值分别为0.071与0.088）。基于CSF3R突变与治疗后MRD将患者分为野生型CSF3R且MRD转阴组、突变型CSF3R或MRD阳性组、突变型CSF3R且MRD阳性组，三组患者RFS（*P*<0.001）与OS（*P*＝0.006）差异均有统计学意义。

**结论:**

CSF3R突变与2个疗程化疗后MRD状态均可预测CEBPA双突变AML患者的长期预后，据此可对患者进行预后再分层。

## Abstract (en)

**Objective:**

This investigation aims to assess the impact of CSF3R mutations and the presence of measurable residual disease（MRD）on the prognosis of patients with CEBPA double mutations who have acute myeloid leukemia（AML）.

**Methods:**

The prognostic significance of these two factors was examined in the present study, which included 66 patients with complete genetic mutations and sequential MRD information.

**Results:**

Following the second course of chemotherapy, the MRD status and CSF3R mutations of these patients were linked to their long-term prognosis. CSF3R mutated patients showed inferior relapse-free survival（RFS）（5-year RFS: 15.2％ *vs* 38.7％, *P*＝0.006）and overall survival（OS）（5-year OS: 18.2％ *vs* 60.6％, *P*＝0.038）compared with those with wild-type CSF3R. After the second course of chemotherapy, patients with negative MRD had an RFS of 64 months and an OS of not reaching, which was significantly longer than that of patients with positive MRD（15 and 48 months, and the *P* value were 0.004 and 0.050, respectively）. CSF3R mutations（*HR*＝0.317, 95％ *CI* 0.129–0.779, *P*＝0.012）, WT1 mutations（*HR*＝0.304, 95％ *CI* 0.115–0.804, *P*＝0.016）, and NRAS mutations（*HR*＝0.153, 95％ *CI* 0.061–0.385, *P*<0.001）were all independently associated with a poor prognosis for RFS, and CSF3R mutations and positive MRD tended to be independently associated with a poor prognosis for OS, according to the results of a Cox proportional-hazards model analysis（*P* values were 0.071 and 0.088, respectively）. The patients were divided into three groups based on their CSF3R mutation status and MRD status following treatment: wide-type CSF3R and negative MRD, mutated CSF3R or positive MRD, and mutated CSF3R and positive MRD, which showed significantly different RFS（*P*<0.001）and OS（*P*＝0.006）.

**Conclusion:**

Both CSF3R mutations and positive MRD were associated with poor outcome in AML patients with CEBPA double mutations. An integrity model based on these two factors may be beneficial for accurately evaluating the prognosis of these patients.

CCAAT增强子结合蛋白α（CCAAT enhancer binding protein α，CEBPα）的编码基因CEBPA突变为急性髓系白血病（AML）常见基因突变之一[1]。CEBPA突变可分为双突变与单突变，多项研究结果表明仅双突变为AML的预后良好指标，而单突变无明显预后意义[2]–[5]。尽管CEBPA双突变AML患者预后良好，文献报道及笔者前期研究结果均显示此类患者的累积复发风险高达30％～50％[6]–[7]。因此，CEBPA双突变AML亦为一组异质性疾病，探索其预后再分层是近年的研究热点之一。

合并基因突变可能是CEBPA双突变AML患者预后再分层的潜在指标，但目前尚存在一定争议[8]–[12]。笔者前期研究发现CSF3R突变为CEBPA双突变AML的不良预后指标之一，突变患者无复发生存（RFS）率与总生存（OS）率均明显低于无突变者[7]。此外，有研究显示微小残留病（MRD）监测在低危组AML患者预后再分层中具有十分重要的指导价值[13]。据此，我们对CSF3R突变和治疗后MRD水平与CEBPA双突变AML患者长期预后的关系进行研究。

## 对象与方法

1. 研究对象：回顾性分析2012年1月至2018年9月就诊于吉林大学第一医院血液科的初诊CEBPA双突变AML患者66例。所有患者均进行细胞形态学、免疫分型、细胞遗传学及基因突变检测，诊断符合文献[1]标准。患者治疗方案见我中心前期报道[7]，初始诱导采用标准剂量“3+7”方案（柔红霉素或去甲氧柔红霉素+阿糖胞苷）、CAG方案（阿糖胞苷+阿克拉霉素+G-CSF）、D-CAG方案（地西他滨+CAG）、MAE方案（米托蒽醌+阿糖胞苷+依托泊苷），缓解后巩固治疗采用大剂量阿糖胞苷（1.5～3.0 g/m^2^）。有适合亲缘或无关供者的部分初诊高白细胞（≥50×10^9^/L）、伴FLT3-ITD突变的第1次完全缓解（CR_1_）患者（2例）及CR_2_患者（4例）行异基因造血干细胞移植。本研究征得患者或家属知情同意并获得吉林大学第一医院伦理委员会批准（2020-556）。

2. 细胞遗传学与基因突变检测：采用常规染色体培养及显带技术分析患者染色体核型，结果描述依据人类细胞遗传学国际命名体系标准[14]。基因突变分析采用二代基因测序技术，具体见我中心前期报道[11]。

3. 流式细胞术检测治疗后MRD水平：患者治疗后采用三激光十色流式细胞仪（Navios，美国贝克曼公司）规律监测MRD，具体方案见我中心前期已发表文献[15]。

4. 疗效评价及随访：1个疗程诱导治疗后评价疗效。具体评估如下：CR为骨髓原始细胞<5％，外周血无原始细胞，无含Auer小体的原始细胞，无髓外白血病，中性粒细胞计数（ANC）≥1.0×10^9^/L且PLT≥100×10^9^/L；CR伴血细胞计数未完全恢复（CRi）为CR伴ANC<1.0×10^9^/L或PLT<100×10^9^/L；部分缓解（PR）为血细胞恢复达CR，骨髓原始细胞比例5％～25％且较治疗前下降至少50％；未缓解（NR）：未达到PR及以上疗效[16]。

所有患者均随访至2021年5月31日。随访方式包括查阅病历、电话。RFS时间定义为CR至复发、死亡或随访截止的时间。OS时间定义为从接受诱导治疗开始至死亡或随访截止的时间。

5. 统计学处理：正态分布的计量资料以均值±标准差表示，非正态分布的计量资料以*M*（*Q*_1_，*Q*_3_）表示。数据分析采用SPSS 20.0软件包。计数资料组间比较采用Fisher精确概率法。Kaplan-Merier法绘制生存曲线，组间比较采用Log-rank检验。将单因素分析中*P*<0.10的指标纳入多因素分析，采用Cox风险比例模型进行多因素分析。*P*<0.05为差异具有统计学意义。

## 结果

1. 患者基线资料：66例患者中男34例，女32例，患者基线资料见表1。绝大部分CEBPA双突变患者FAB分型为M_2_亚型（62.12％，41/66），染色体核型正常（90.38％，47/52）。异常染色体核型患者共5例，包括del（9q）2例，+8、+21、+Y各1例。

**表1:** 66例CEBPA双突变急性髓系白血病患者基本临床资料

临床特征	数值
年龄[*M*（范围）]	44.5（9~79）
性别[例（%）]	
男	34（51.52）
女	32（48.48）
FAB分型	
M_1_	3（4.55）
M_2_	41（62.12）
M_4_	15（22.73）
M_5_	4（6.06）
M_6_	3（4.55）
细胞遗传学^a^[例（%）]	
正常核型	47（90.38）
异常核型	5（9.62）
WBC[×10^9^/L，*M*（*Q*_1_，*Q*_3_）]	19.24（8.34, 58.26）
HGB（g/L, *x±s*）	97.62 ± 24.48
PLT[×10^9^/L，*M*（*Q*_1_，*Q*_3_）]	22.50（12.00, 42.00）
骨髓原始细胞比例（%, *x±s*）	61.82 ± 18.94
初始诱导方案[例（%）]	
DA方案	19（28.79）
IA方案	43（65.15）
其他	4（6.06）
异基因造血干细胞移植[例（%）]	6（9.68）

注 DA：柔红霉素+阿糖胞苷；IA：去甲氧柔红霉素+阿糖胞苷。^a^14例患者染色体检测时未找到分裂象

2. CEBPA双突变患者合并基因突变：采用二代基因测序共检出32种合并基因突变，其中发生率最高的3种突变基因分别为WT1（28.79％，19/66）、CSF3R（24.24％，16/66）与NRAS（19.70％，13/66）。本组患者未检出NPM1突变。发生率>5％的合并基因突变见图1。

**图1:**
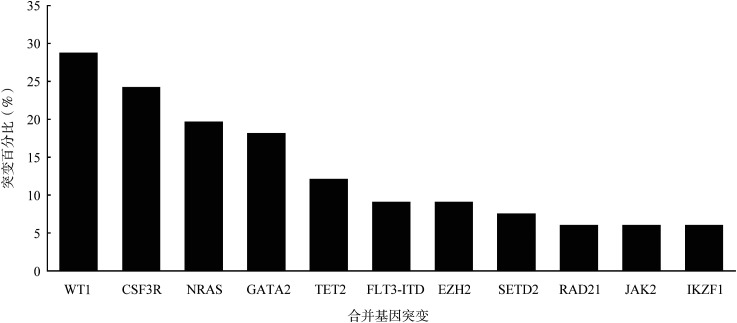
66例CEBPA双突变急性髓系白血病患者常见合并基因突变

3. 疗效分析：所有患者经1个疗程诱导化疗后，59例（89.39％）获得CR，6例（9.10％）获得PR，1例NR。2个疗程后所有患者均获得CR。中位随访时间为18（4～78）个月，1、3、5年的RFS率分别为92.0％、62.5％和42.6％，1、3、5年的OS率分别为96.7％、88.7％和50.8％。

CSF3R突变患者的5年RFS率仅为15.2％，明显低于无突变患者的38.7％，中位RFS时间分别为11个月和64个月（*P*＝0.006）（图2A）。CSF3R突变患者与无突变患者的5年OS率分别为18.2％和60.6％，中位OS时间分别为27个月和未达到（*P*＝0.038）（图2B）。1个疗程获得CR（包括CRi）与患者长期预后无关（*P*值均>0.05）（图3A、3B）。2个疗程治疗后流式细胞术MRD状态与患者长期预后明显相关。MRD阴性患者的RFS、OS均明显优于阳性患者，中位RFS时间分别为64个月和15个月（*P*＝0.004）（图4A），中位OS时间分别为未达到和48个月（*P*＝0.050）（图4B）。

**图2:**
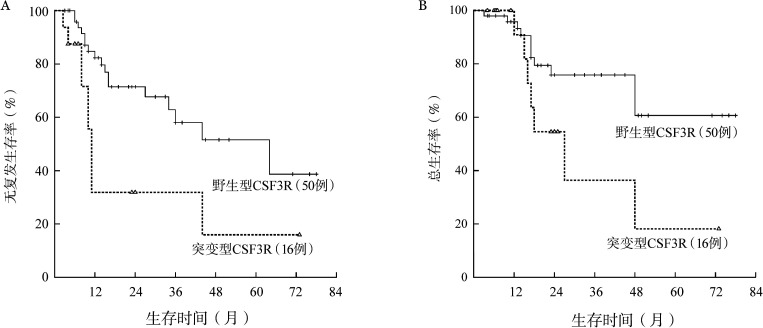
CSF3R突变对CEBPA双突变急性髓系白血病患者无复发生存（A）和总生存（B）的影响

**图3:**
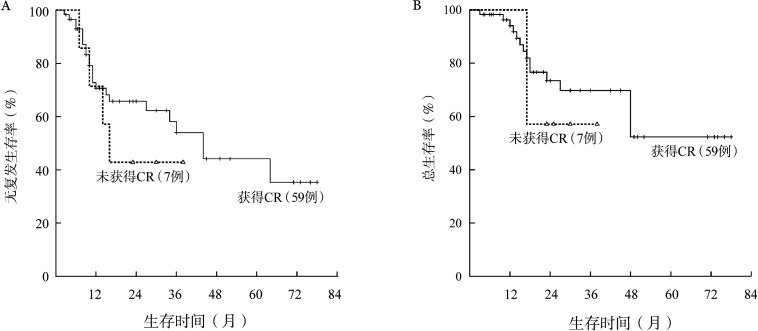
1个疗程是否获得完全缓解（CR）对CEBPA双突变急性髓系白血病患者无复发生存（A）和总生存（B）的影响

**图4:**
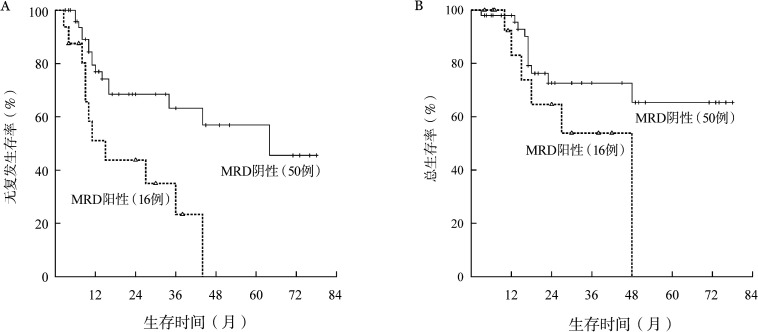
2个疗程化疗后微小残留病（MRD）状态对CEBPA双突变急性髓系白血病患者无复发生存（A）和总生存（B）的影响

4. Cox风险比例模型分析影响CEBPA双突变AML患者预后的指标：单因素分析显示CSF3R突变（*P*＝0.009）、WT1突变（*P*＝0.022）、NRAS突变（*P*＝0.001）和MRD阳性（*P*＝0.006）与RFS显著相关，而CSF3R突变（*P*＝0.051）和MRD阳性（*P*＝0.064）趋向与OS有关（表2）。多因素分析示CSF3R突变（*P*＝0.012）、WT1突变（*P*＝0.016）、NRAS突变（*P*<0.001）为影响患者RFS的独立不良预后因素，而CSF3R突变（*P*＝0.071）与MRD阳性（*P*＝0.088）趋向于为OS的不良预后因素（表3）。

**表2:** 影响CEBPA双突变急性髓系白血病患者生存的单因素分析

参数	RFS			OS		
	风险比	95% *CI*	*P*值	风险比	95% *CI*	*P*值
性别（男/女）	0.944	0.443~2.015	0.883	0.940	0.372~2.374	0.896
年龄	0.982	0.953~1.013	0.253	1.005	0.971~1.041	0.770
染色体核型（正常/异常）	2.113	0.280~15.936	0.468	23.369	0.013~42 369.939	0.410
初诊WBC	1.001	0.994~1.007	0.817	1.001	0.993~1.009	0.822
CSF3R突变	0.349	0.159~0.769	0.009	0.388	0.013~1.005	0.051
WT1突变	0.396	0.179~0.877	0.022	0.573	0.215~1.531	0.267
NRAS突变	0.221	0.094~0.518	0.001	0.480	0.156~1.477	0.201
GATA2突变	0.994	0.379~2.255	0.902	1.024	0.334~3.137	0.967
TET2突变	0.987	0.337~0.893	0.981	1.590	0.365~6.923	0.537
MRD阳性	0.333	0.152~0.730	0.006	0.407	0.157~1.053	0.064

注 MRD：微小残留病；RFS：无复发生存；OS：总生存

**表3:** 影响CEBPA双突变急性髓系白血病患者生存的多因素分析

参数	RFS			OS		
	风险比	95% *CI*	*P*值	风险比	95% *CI*	*P*值
WT1突变	0.304	0.115~0.804	0.016			
NRAS突变	0.153	0.061~0.385	<0.001			
CSF3R突变	0.317	0.129~0.779	0.012	0.413	0.158~1.077	0.071
MRD阳性	0.562	0.226~1.401	0.216	0.434	0.166~1.133	0.088

注 MRD：微小残留病；RFS：无复发生存；OS：总生存

CSF3R突变患者中，56.25％（9/16）的患者在2个疗程化疗后获得MRD转阴，而野生型CSF3R患者MRD转阴率为82.00％（41/50，*P*＝0.049）。考虑到CSF3R突变与MRD状态的密切相关性，我们基于CSF3R突变与2个疗程后MRD状态将患者再分层：野生型CSF3R且MRD转阴组、突变型CSF3R或MRD阳性组、突变型CSF3R且MRD阳性组。结果可见三组患者RFS（*P*<0.001）与OS（*P*＝0.006）差异均有统计学意义（图5A、5B）。

**图5:**
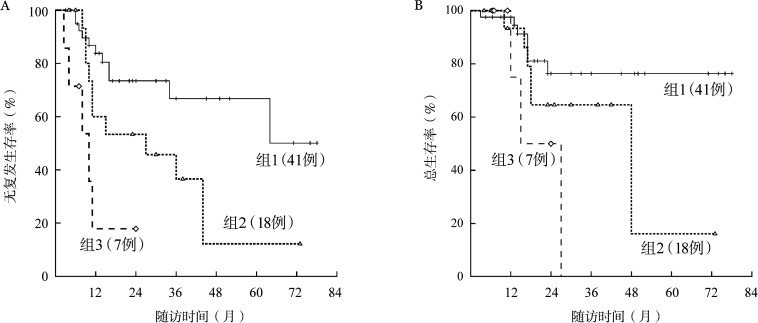
基于CSF3R突变与2个疗程后微小残留病（MRD）状态对CEBPA双突变急性髓系白血病患者再分层后生存的比较 A：三组无复发生存的比较；B：三组总生存的比较。组1：野生型CSF3R且MRD转阴组；组2：突变型CSF3R或MRD阳性组；组3：突变型CSF3R且MRD阳性组

## 讨论

近年来，越来越多研究结果显示CEBPA双突变AML为一组异质性的疾病[6]–[7],[12],[17]。因而，多项研究尝试对此类患者进行预后再分层，其中合并基因突变与治疗后MRD可能是潜在指标[7]–[12],[17]。本研究通过整合治疗前合并基因突变与治疗后化疗敏感性指标MRD综合判断患者预后，建立新的预后预测模型，进而对此类患者进行预后再分层。

我中心曾报道了CEBPA双突变AML患者的基于二代基因测序技术的基因突变谱特征[11]，结果发现可能具有预后再分层价值的共突变基因包括GATA2、WT1、TET2与CSF3R[7],[11]。Fasan等[8]报道GATA2突变是CEBPA双突变AML患者OS的预后良好指标，但其对无事件生存（EFS）无明显影响。然而，多项研究均未观察到上述现象[9]–[11]。有文献报道WT1基因突变为正常核型成人AML患者的不良预后指标之一[18]。在CEBPA双突变AML中，与野生型基因患者相比，WT1突变患者具有更短的OS时间（中位时间为14个月与未达到）与无病生存时间（中位时间为7.8个月与未达到）[12]。本组数据结果显示WT1突变（*P*＝0.022）与NRAS突变（*P*＝0.001）为患者RFS的独立不良预后指标，但其对OS均无明显影响（均*P*>0.05）。有研究报道TET2突变患者比野生型基因患者具有更低的1年OS率（分别为78.3％和94.3％）与EFS率（分别为53.8％和72.6％）[19]。然而，在对中国人群的研究中未得出上述结论[11]–[12]。在成人及儿童AML患者中均观察到CSF3R突变与CEBPA双突变密切相关[20]，笔者首次证实CSF3R突变与患者不良预后显著相关[7]。近来国内学者采用线列图建立了CEBPA双突变AML患者新的预后预测模型，而CSF3R突变是其中重要参数之一[21]–[22]。综上，目前研究结果提示合并基因突变对CEBPA双突变AML患者的预后意义尚存在一定争议。然而，分析上述结果时需考虑以下因素。首先，种族因素导致基因遗传背景的差异可能会影响不同基因突变的预后意义。其次，上述研究纳入的患者数均较少，尚待扩大样本进一步研究。笔者曾试图采用荟萃分析在大样本中比较不同基因突变的预后意义，但由于异质性过大而未能合并。最后，CEBPA双突变AML是化疗敏感性疾病，不同中心治疗方案不同，可能在一定程度上影响了合并基因突变的预后意义。

MRD水平可反映化疗敏感性，与AML患者的长期预后密切相关[23]。通过多参数流式细胞术检测巩固治疗过程中MRD水平发现：与MRD阴性患者相比，MRD阳性患者具有更高的复发风险与更低的RFS率（3年RFS率分别为45.0％和63.3％, *P*＝0.037）[24]。北京大学人民医院根据巩固治疗过程中MRD水平将患者分为高危组与低危组，前者为2个疗程巩固治疗后MRD仍为阳性或MRD由阴性转为阳性，后者为MRD持续阴性，结果显示MRD危险分组为患者长期预后的独立影响因素之一[17]。近来，我国多中心临床研究结果再一次证实，治疗后MRD危险分组与患者长期预后密切相关[25]。因此，巩固治疗中MRD水平是CEBPA双突变AML患者重要的预后指标之一。

本研究中我们发现CSF3R突变与2个疗程化疗后MRD状态均可预测患者的长期预后。多因素分析结果显示CSF3R突变、WT1突变与NRAS突变为RFS的独立预后因素，CSF3R突变与MRD阳性趋向于为OS的独立预后因素。考虑到CSF3R突变与MRD状态的密切相关性，我们提出一个新的基于CSF3R突变与MRD状态的CEBPA双突变AML患者预后分层模型，可将患者分为不同危险组。尽管我们的预后模型建立在相对小样本的基础上，尚需扩大样本或前瞻性临床试验验证，但该模型综合了治疗前与治疗后参数，对后续研究预测模型的建立具有一定参考意义。

综上所述，CEBPA双突变AML为一异质性疾病群体。治疗前基因突变与治疗后化疗敏感性指标可能用于此类患者预后再分层，基于CSF3R突变与MRD状态可建立新的综合预后预测模型。此外，考虑到种族基因遗传背景与不同中心治疗方案的差异，在后续研究中，CSF3R可能更换为其他突变基因，进而联合MRD状态更好地评估CEBPA双突变AML患者的预后。因而，本研究结果可能为优化此类患者的临床管理提供有价值的参考。
